# Investigation of *cgrA* and *cyp51A* gene alternations in *Aspergillus fumigatus* strains exposed to kombucha fermented tea

**DOI:** 10.18502/cmm.5.3.1745

**Published:** 2019-09

**Authors:** Ladan Nazemi, Seyed Jamal Hashemi, Roshanak Daie Ghazvini, Mina Saeedi, Sadegh Khodavaisy, Aleksandra Barac, Mona Modiri, Maryam Akbari Dana, Zahra Zare shahrabadi, Sassan Rezaie

**Affiliations:** 1 Division of Molecular Biology, Department of Medical Mycology and Parasitology, School of Public Health, Tehran University of Medical Sciences, Tehran, Iran; 2 Medicinal Plants Research Center, Faculty of Pharmacy, Tehran University of Medical Sciences, Tehran, Iran; 3 Persian Medicine and Pharmacy Research Center, Faculty of Pharmacy, Tehran University of Medical Sciences, Tehran, Iran; 4 Department of Medical Biotechnology, School of Advanced Technologies in Medicine, Tehran University of Medical Sciences, Tehran, Iran; 5 Clinic for Infectious and Tropical Diseases, Clinical Center of Serbia, Faculty of Medicine, University of Belgrade, Belgrade, Serbia

**Keywords:** Aspergillus fumigatus, CgrA gene, Cyp51A gene, Kombucha

## Abstract

**Background and Purpose:**

*Aspergillus fumigatus* is one of the most common opportunistic fungus, which causes infection in immunocompromised and neutropenic patients. The current guidelines recommend voriconazole as the initial therapeutic and prophylactic agent for almost all cases, especially in patients with organ transplants, which leads to increased medication resistance in *A. fumigatus*. The aim of the present study was to evaluate the antifungal activity and effect of kombucha as a natural compound on *A. fumigatus* growth, as well as on the expression of *cgrA* and *cyp51A* genes.

**Materials and Methods:**

A panel of 15 *A. fumigatus *strains with two quality controls of CM237 and CM2627 as susceptible and resistant strains were obtained from Tehran Medical Mycology Laboratory, Tehran,Iran(TMML).Antifungal susceptibility testing assay was performed according to the Clinical and Laboratory Standards Institute (CLSI) M38-A2 document. Moreover, the mycelial dry weight of the fungus was calculated before and after being treated with kombucha. In addition, the quantitative changes in the expression of* cgrA* and *cyp51A* genes were analyzed by real-time polymerase chain reaction (real-time PCR) technique.

**Results:**

In the present study, the minimum inhibitory concentration ranges of kombucha were measured at 6,170 and 12,300 μg/mL for ten *A. fumigatus* azole-susceptible strains and 24,700 μg/mL for five *A. fumigatus* resistant strains. Moreover, changes in mycelial dry weight under kombucha treatment conditions underwent a significant reduction (*P≤0.05*). A coordinate down-regulation of expression in* cgrA* and *cyp51A* genes was observed in all azole-susceptible and -resistant *A. fumigatus *strains, after treating the fungus with different concentrations of kombucha (*P≤0.05*).

**Conclusion:**

According to the obtained results, kombucha as a natural antioxidant , can exert inhibitory effects against the growth and expression of some genes in *A. fumigatus*strains.

## Introduction

*Aspergillus fumigatus* is a ubiquitous filamentous fungus that produces billions of spores, which are unavoidably inhaled on a daily basis [1]. *A. fumigatus* is associated with a range of localized and invasive diseases [2]. There is a dramatic increase in the incidence of invasive aspergillosis (IA) among immunocompromised patients [3, 4]. The pathogenicity of *A. fumigatus* is affected by various virulence factors. One of the most important virulence factors is the ability to grow well at 37°C and tolerate high temperatures [4, 5]. Thermometric property is affected by some important gene functions (e.g., *cgrA*) and heat shock protein 90 family (e.g., Hsp1 and Asp f12) [4, 5]. 

In the past 15 years, triazole compounds were considered as the first choice for the prevention and treatment of aspergillosis [6]. Consequently, resistance to azoles emerges as a global health issue, and it is increasingly clear that patients with azole-resistant *A. fumigatus* are at a higher risk of failure in therapy [7]. Azole resistance is often associated with key mutations, including TR34/L98H, TR53, and TR46/Y121F/T289A or the overexertion of the *cyp51A* gene [8]. The majority of the cases with azole-resistant disease are caused due to resistant *A. fumigatus*, which is originated from the use of agricultural fungicides in the environment [9]. 

Clearly, it is important to investigate the development of new antifungal compounds using traditional food and beverages for the growth inhibition of *A. fumigatus*. The symbiosis culture of bacteria and yeast (SCOBY) which is known as “Kombucha” is an oriental beverage obtained from the fermentation of sugared tea [10]. It contains organic acids, active enzymes, vitamins, amino acids, and polyphenols produced during the fermentation process [10]. The antimicrobial effects of kombucha as a natural compound and powerful antioxidant agent has been proved [11-16]. However, the mechanism of kombucha in dealing with *A. fumigatus *strains is not clear yet. Regarding this, the aim of the present study was to investigate the inhibitory effect of kombucha fermented tea against *A. fumigatus* growth and its possible impact on the expression of *cgrA* and *cyp51A* genes.

## Materials and Methods

***Strains***

A panel of 15 *A. fumigatus* strains, including ten azole-susceptible species and five azole-resistant strains, were obtained from the strain collection of Tehran Medical Mycology Laboratory (TMML), Tehran, Iran. A wild-type *A. fumigatus* (CM237) and a triazole-resistant strain (CM2627) carrying the TR34/L98H mutation were used as controls. The strains were identified as *A. fumigatus* by macro- and micro-morphology under the biosafety condition of level II. This was also confirmed by the gene sequencing of beta-tubulin and calmodulin. All strains were subcultured on Sabouraud dextrose agar medium for 2 days at 35°C. 

***Preparation of Kombucha Tea***

The infusions were prepared after mixing 20 g of black tea leaves and 80 g of white sugar in 1 L of boiling water and steeping it for 15 min. Then, the infusions were filtered using filter papers. The resultant clear filtrate was poured into 2500-mL Erlenmeyer flasks. Then, it was cooled to room temperature; subsequently, 10% (w/v) kombucha starter tea was added into the flasks. Next, the darker side of the SCOBY was put on the sweet tea solution. The flasks were then covered and incubated at room temperature for 14 days.

***Antifungal Susceptibility Testing***

Antifungal susceptibility testing was performed using a modified microbroth dilution method in accordance with the Clinical and Laboratory Standards Institute M38-A2 protocol [18]. To determine the antifungal activities of kombucha against *A. fumigatus* strains, they were tested at a kombucha final concentration range of 395,500-770 µg/mL. To this end, they were dispensed into 96-well microdilution trays (Suzhou Conrem Biomedical Technology Co., China) using RPMI 1640 medium (Sigma-Aldrich, St. Louis, MO, USA) buffered with MOPS (Sigma-Aldrich, St. Louis, MO, USA). 

Thereafter, fungal spores were harvested from a two-day culture (in the logarithmic growth phase) and suspended in distilled water containing 0.05% Tween-40 (Sigma-Aldrich, St. Louis, MO, USA). *Aspergillus* spores were counted on a hemocytometer and the optical density (OD) was adjusted at a final concentration of 0.5-4×10^4^ CFU/mL (80-82%T; OD: 0.09-0.13). Next, 0.1 mL of the working inoculum was added to the microdilution plates, which were incubated at 35°C and examined visually for the determination of minimum inhibitory concentrations (MICs) within 48 h of incubation. The growth of the organism was considered if the test sample grew more than the positive control. In addition, unchanged growth was suggestive of the ineffectiveness of kombucha. In contrast, 90% growth inhibition of fungal elements indicated the lack of fungal growth and the positive inhibitory effect of kombucha at the studied concentration. The MIC endpoints were determined using a mirror and defined as the lowest concentration of the antifungal drug or kombucha that significantly reduced growth in comparison with the positive control. 

Voriconazole powder (Sigma-Aldrich, St. Louis, MO, USA) was dissolved in dimethyl sulfoxide and diluted in standard RPMI 1640 medium (Sigma, St. Louis, MO, USA) buffered at pH 7.0 with 0.165 mol.L [3-(N-morpholino) propanesulfonic acid] buffer with L-glutamine without bicarbonate (MOPS, Sigma, Louis, MO,USA). The strains with the MICs of >1 µg/mL for itraconazole and voriconazole and ≥ 0.5 µg/mL for posaconazole were considered as non-wild-type [17]. Control strains, namely *Paecilomyces variotii *(ATCC 22319), were used in each test. In addition, positive control (without any sample solution) and negative controls (without any fungal inoculum) were prepared. The minimum fungicidal concentration (MFC) of kombucha was obtained by the absence of colonies in the plate. 

***Mycelial Dry Weight/Biomass Production***

Fungal mycelium was prepared in two groups, including the test group (treated with kombucha) according to MIC results and control group (untreated with kombucha), in a massive volume. Then, fungal mycelia were harvested after 48 h of growth and separated from the liquid culture RPMI 1640 (Sigma, St. Louis, MO, USA) by filtration through a Whatman filter paper No.1 (Sigma-Aldrich, Germany). Subsequently, mycelial mass was repeatedly washed with distilled water and dried overnight at 50°C. The fungus dry weight before and after being treated with kombucha was calculated using the following formula: 

DW= (W2-W1) 

Where DW represents dry weight of mycelium mat, W2 signifies the weight of test fungus along with filter paper, and W1 denotes the weight of filter paper. All the tests were performed twice.

***High-Performance Liquid Chromatography Method***

***Determination of Acetic Acid***

High-performance liquid chromatography (HPLC) analysis was performed on samples fermented for 14 days using a Shimadzu DGU-28 series liquid chromatograph system, equipped with a SPD-20A photodiode array detector set to record at 210 nm . The samples were separated with a phenomenex C18 reverse-phase column (5 µm, 150×4.6 mm) using a low-pressure gradient mobile phase of 0.1% H_3_PO_4 _in H_2_O with a 20-40-20 MeOH gradient (20%-40% MeOH from t=0-6 min, 40%-20% from t=6-9 min, hold at 20% MeOH for 1 min). The flow rate was 0.75 mL/min, and the total run-time was 10 min. The column was operated at room temperature, and the sample injection volume was 20 µL. Standard additions were prepared for acetic acid and glucoronic acid (Sigma-Aldrich, Germany). All the samples were filtered prior to HPLC analysis using a 0.45-µm filter.

***Determination of Water-Soluble Vitamins (Thiamine and Ascorbic Acid)***

The HPLC-grade solvents were used for analysis. Analytical reagent-grade acetonitrile and methanol (Tedia Company, USA). The water used for HPLC and sampling was prepared with a Millipore Simplicity instrument (Millipore, Molsheim, France). All the vitamin standards were of chromatography grade and were purchased from Sigma Chemical Co. (Poole, Dorset).

For thiamine_,_ kombucha tea (100 mL) was placed in 25 mL of H_2_SO_4_ (0.1 N) solution and incubated for 30 min at 121°C. Then, the contents were cooled and adjusted at a pH of 4.5 with 2.5 M sodium acetate; subsequently, 50 mg Takadiastase enzyme was added. The preparation was stored overnight at 35°C. The mixture was then filtered through a Whatman No. 4 filter; in the next stage, it was diluted with 50 mL pure water and re-subjected to filtration through a micropore filter (0.45 μm). 

Finally, 20 μL of the filtrate was injected into the HPLC system. The quantification of thiamine content was accomplished by comparing it with thiamine standards. Chromatographic separation was achieved on a reversed phase-(RP-)HPLC column (Agilent ZORBAX Eclipse Plus C18; 250×4.6 mm i.d., 5 μm) through the isocratic delivery mobile phase (A/B 33/67; A: MeOH, B: 0.023 M H_3_PO_4_, pH=3.54) at a flow rate of 0.5 mL/min. Ultraviolet (UV) absorbance was recorded at 270 nm at room temperature. 

For ascorbic acid detection, the kombucha tea (100 mL) was added to the extracted solution containing metaphosphoric acid (0.3 M) and acetic acid (1.4 M). The mixture was placed in a conical flask and agitated at 10,000 rpm for 15 min. The mixture was then filtered through a Whatman No. 4 filter, and the samples were extracted in triplicate. The ascorbic acid standard was prepared by dissolving 100 mg of L-ascorbic acid in a metaphosphoric acid (0.3 M)/acetic acid (1.4 M) solution at a final concentration of 0.1 mg/mL. The calibration line was converted to a linear range based on four measured concentration levels.

Quantification of ascorbic acid content was performed on an Agilent HPLC system. Chromato-graphic separation was achieved on an RP-HPLC column through the isocratic delivery of a mobile phase (A/B 33/67; A: 0.1 M potassium acetate, pH=4.9, B: acetonitrile: water [50: 50]) at a flow rate of 1 mL/min. The UV absorbance was recorded at 254 nm at room temperature.

***RNA Extraction and Complimentary complementary DNA Synthesis***

After 48 h of incubation at 35°C, total RNA was extracted from *A. fumigatus* strains under both kombucha-treated (24,700 , 12,300 and 6,170 µg/mL) and -untreated conditions as control. Afterwards, the harvested mycelia were separately collected for each sample in 15-cc sterilized falcon tube (Ariantajhiz, Iran), then centrifuged for 10 min and vortexed for 5-10 sec. Sedimentation of the specimens was transferred to the microtube, and total RNA was extracted using the RNX-plus Kit (Sinacolon, Karaj, Iran) following the instructions of the manufacturer. 

The pellet was then rinsed with cold ethanol and incubated for 10 min at 55-60°C in Ben-Marie. The RNA concentration and purity were determined spectrophotometrically (Eppendorf, Germany Biopho-tometer), and equal concentration of RNA (1µg in 30 mL) was subjected to complementary DNA (cDNA) synthesis using the PrimeScript RT reagent kit (Parstous, Iran).

***Primer Designing***

The primers used for testing the above-mentioned gene by real-time PCR were designed using PRIMER3 web-based software (http://bioinfo.ut.ee/primer 3-0.4.0) as shown in Table1. The *β-actin* gene was used as the endogenous reference gene. The primers were checked for specificity through the BLASTN search based on the National Center for Biotechnology Information website (http://blast.ncbi.nlm.nih.gov/blast.cgi).

***Real-time*** ***Polymerase Chain Reaction***

The changes in the expression of *cgrA* and *cyp51A *genes were studied in the azole-susceptible and -resistant *A. fumigatus *strains by quantitative real-time PCR. Real-time PCR reaction was performed with Corbett rotor-gene 2.1.0.9 (QUIAGEN, US) according to an optimized three-step cycling protocol. The amplification process was monitored using the 2X real-time PCR master mix including SYBR^®^ Green kit (Biofact, Korea). For each reaction, 1 µL of each primer and 2 µL of cDNA were added. The final volume was adjusted to 20 µL with DEPC-treated water. The real-time PCR program conditions for *cgrA* and *cyp51A *detection consisted of initial denaturation at 95°C for 15 min, followed by 20 sec at 95°C, 20 sec at 60°C, and 30 sec at 72°C according to the 2X real-time PCR master mix kit (Biofact, Korea) protocols. 

**Table1:** Primers used in real-time polymerase chain reaction

**Accession No**	**Name**	**nt**	**GC(%)**	**Tm**	**Sequence(5'-3')**
AY008837.1	*cgrA-F*	20	55	59.67	5'- GGCAGGAGGCAGTTAAGGAA-3'
AY008837.1	*cgrA-R*	20	55	60.04	5'-TGAAGCAACAGCG GAGAGAG-3'
MH781385.1	*Cyp51A-F*	20	43	55.92	5'-CATGTGCCACTTATTGAGAAG-3'
MH781385.1	*Cyp51A-R*	20	48	57.87	5'-TTGATGGGAGTAAAGCCCTTG-3'
MH716022.1	*βact- F*	20	55	60.39	5'-TGAGAGGGAAATCGTGCGTG-3'
MH716022.1	*βact- R*	21	52.38	61.29	5'-TGCTTGCTGATCCACATCTGC-3'

Each test performed was in triplicate, and the mean values of the relative expression were determined for *cgrA* and *cyp51A* genes. To confirm the specificity of amplification, the amplified fragments were analyzed by electrophoresis on 2% agarose gel containing ethidium bromide. The expression of all genes was normalized to the housekeeping gene, and all the data were analyzed using REST software (2009) with an accuracy of 95% (*P≤ 0.05*). This software applies the comparative 2^-ΔΔCt^ method. The *CT* is the average threshold of the cycle from three independent experiments. The reaction efficiency of all samples was analyzed by the REST software. 

***Statistical Analysis***

All the data were statistically analyzed in SPSS software (version 16; IBM Corp., Armonk, NY) by the paired t-test and Fisher’s exact test. A *P-value of ≤ 0.05* was considered statistically significant.

## Results

The effects of different concentration of kombucha (395,500-770 μg/mL) was investigated following 48 h of incubation at 35°C. In addition, voriconazole (16-0.03125 μg/mL) was used for qualitative comparison. According to our results, the MIC ranges of kombucha were 6,170-12,300 μg/mL for ten *A. fumigatus* azole-susceptible strains (voriconazole MIC range: 1-0.125 μg/mL) and 24,700 μg/mL for five *A. fumigatus* resistant strains (voriconazole MIC range: 16-4 μg/mL). Moreover, out of a total of ten azole-susceptible strains of *A. fumigatus*, the MFC values of kombucha were reported as follows: 24,700 μg/mL in six strains, 12,300 μg/mL in three strains,and 49,400 μg/mL in one strain (voriconazole MFC range: 2-0.25 μg/mL). 

Furthermore, the MFCs rate of kombucha in *A. fumigatus*-resistant strains was 49,400 μg/mL in three strains and 98,800 μg/mL in two strains (voriconazole MFC range: 32-8 μg/mL). Table 2 summarizes the MFCs for voriconazole and kombucha tested in azole-susceptible and -resistant *A. fumigatus* strains. In order to quantitatively compare the efficacy of kombucha in inhibiting the growth of *A. fumigatus* strains, the mycelial dry weight was calculated. 

With regard to azole-susceptible *A. fumigatus*, the fungal mycelial weights were estimated at 0.08-0.2 and 1.23-1.29 g after and before treatment with kombucha, respectively. On the other hand, the mycelial weights of azole-resistant strains were obtained as 1.26-1.29 and 0.08-0.13 g prior to and following the treatment with kombucha, respectively. The analysis showed that the pre- and post-treatment of mycelial weight were different in both groups with a p-value of ≤ 0.05 (Table 3). The results obtained from the HPLC are summarized in Table 4.

**Figure 1:**
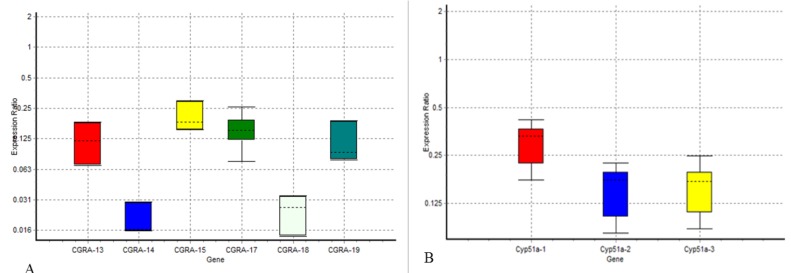
Ratio of the expressions of genes (A) *cgrA *and (B) *cyp51A* (Boxes represent the interquartile range or the middle 50% of observations. The dotted line signifies the median gene expression. Whiskers denotes minimum and maximum observations.)

Real-time PCR was performed using the SYBR Green method. Significant and proportional down-regulation of *cgrA* and *cyp51A* genes was observed in all *A. fumigatus* strains after being treated with 6,170 and 12,300 μg/mL concentrations of kombucha for azole-susceptible strains and 24,700 μg/mL for resistant strains (*P≤0.05*). The* cgrA* gene was down-regulated (0.115, 0.019, 0.152, and 0.023 folds) in four susceptible *A. fumigatus* strains (i.e., TMML 026, TMML 027, TMML 014, and TMML 585) and (0.204 to 0.110-fold) in two resistant *A. fumigatus* strains (i.e., TMML 025 and TMML 566). The* cyp51A* genes were down-regulated (0.295- to 0.147-fold) in two susceptible *A. fumigatus* strains (i.e., TMML 026 and TMML027) and in TMML 025 resistant *A. fumigatus* strain (0.154-fold). Table 5 demonstrates the gene expression rates in azole-susceptible and -resistant *A. fumigatus* strains. Figure 1 indicates the ratio of expression under the concentration of kombucha-treated and -untreated conditions.

**Table 2:** The minimum inhibitory concentrations and minimum fungicidal concentrations of voriconazole and kombucha against azole-susceptible and -resistant *Aspergillus fumigatus *strains

**Strain No**	**Voriconazole** **MIC (µg/mL)**	**Voriconazole** **MFC(µg/mL)**	**Kombucha** **MIC(µg/mL)**	**Kombucha** **MFC(µg/mL)**
CM237	0.125	0.25	6,170	12300
TMML 026	0.125	0.25	12,300	24,700
TMML 013	0. 5	1	12,300	24,700
TMML 022	0.125	0.5	12,300	24,700
TMML 027	0.125	0.25	6,170	12,300
TMML 014	0.25	0.5	12,300	24,700
TMML 747	0. 5	1	12,300	24,700
TMML 585	0.25	0.5	6,170	12,300
TMML 601	0.125	0.25	6,170	12,300
TMML 587	0.125	0.5	12,300	49,400
TMML 590	1	2	12,300	24,700
CM2627	4	8	24,700	49,400
TMML 025	4	8	24,700	98,800
TMML 610	8	16	24,700	49,400
TMML 566	4	8	24,700	49,400
TMML 608	4	8	24,700	98,800
TMML 603	16	32	24,700	49,400

MIC: Minimum Inhibitory Concentration, MFC: Minimum Fungicidal Concentration

**Table 3:** Mycelial dry weight of *Aspergillus* *fumigatus* strain in RPMI 1640 medium

**Kombucha (µg/mL)**	Mycelial dry weight (g)	
	**Test group** **(Treated with kombucha)**	**Control group** **(Untreated with kombucha)**
6,170	0.08-0.18	1.24-1.28
12,300	0.08-0.2	1.23-1.29
24,700	0.08-1.13	1.26-1.29

**Table 4:** Results of high-performance liquid chromatography analysis of kombucha

**No**	**Chemical Compounds**	**Result**	**Unit**
1	Acetic acid	61.8	mg/L
2	Ascorbic acid	1.8	mg/100mL
3	Thiamine	0.42	mg/100mL

**Table 5:** Gene expression rates in azole-susceptible and -resistant *Aspergillus fumigatus *strains

**Strain No**	**Gene**	**Type**	**Reaction efficiency**	**Expression**	**Std. Error**	**95% C.I.**	**P(H1)**	**Result**
	*β act*	REF	1.0	1.000				
TMML 026	*cgrA-13*	TRG	1.0	0.115	0.069 - 0.184	0.069 - 0.184	0.000	DOWN
TMML 027	*cgrA-14*	TRG	1.0	0.019	0.015 - 0.030	0.015 - 0.030	0.028	DOWN
TMML 025	*cgrA-15*	TRG	1.0	0.204	0.154 - 0.299	0.154 - 0.299	0.028	DOWN
TMML 014	*cgrA-17*	TRG	1.0	0.152	0.116 - 0.229	0.083 - 0.254	0.022	DOWN
TMML 585	*cgrA-18*	TRG	1.0	0.023	0.014 - 0.034	0.014 - 0.034	0.000	DOWN
TMML 566	*cgrA-19*	TRG	1.0	0.110	0.077 - 0.188	0.077 - 0.188	0.028	DOWN

P(H1): probability of alternate hypothesis that difference between sample and control groups is only due to chance, TRG: target

## Discussion

In recent years, IA has remained a major burden of infectious disease among the immunocompromised population in developing countries [4, 19-21]. The current guidelines recommend voriconazole as the initial therapeutic and prophylactic agent for almost all patients, especially for the cases with organ transplantation experience [22]. Nowadays, voriconazole resistance has led to failure in IA therapy [23, 24]. 

 Therefore, it is required to develop high-potential natural antifungals due to the increase of antifungal drug resistance in *A. fumigatus, *side effects of medications, pharmacological interactions, costly medications, and sometimes the unavailability of some agents [25]. Kombucha tea with unique aromatic, sweet, and sour taste contains various types of vitamins, minerals, and organic acids of fermented tea leaves and produced from the fermentation of tea and sugar [15, 16, 26-33]. Kombucha as a natural product has strong antioxidant, antimicrobial, antiviral, and anticancer properties [34]. However, the mechanism of the inhibitory effect of kombucha on fungal growth is not sufficiently investigated. Subsequently, the present study brought new knowledge about the antifungal impact of kombucha, which could be a potent antifungal agent.

According to the related literature, kombucha tea has no significant toxicity [15, 16]. Vijayaraghavan et al. (2000) indicated that rats fed with kombucha tea for 90 days did not show any signs of toxic effects. Pauline et al. (2001) studied the toxicity of kombucha tea through oral feeding of rats for 15 days at three different doses of kombucha tea. These authors did not report any remarkable toxicity for this tea [15, 16]. 

Mahmoudi et al. (2016) examined the influence of kombucha tea ethyl acetate on *Malassezia* species isolated from the skin with seborrheic dermatitis. Based on their results, kombucha fermented tea showed an inhibitory effect against *Malassezia* species. The mentioned authors observed the lowest and highest MICs for* Malassezia slovakia* and *M. restricta,* respectively [13].

Al-Kalifawi et al. (2014) investigated the antimicrobial property of kombucha tea against bacteria isolated from diabetic foot ulcer. They showed kombucha tea to have a potent antibacterial impact [27]. Ari Yuniarto et al. (2009) indicated that kombucha tea has a considerable antifungal effect against human pathogenic fungi, *such as* A. flavus, Candida albicans, *and* Microsporum gypseum *[*34*]. *

*A. fumigatus *is able to grow optimally under the conditions of thermal stress due to the presence of nucleolar protein *cgrA. *This gene is required to support rapid growth as the characteristic of *A. fumigatus *at a high temperature. In a study performed by Ruchi Bhabhra et al. (2006), a mutant lacking *cgrA* grew normally at 22°C, grew slowly at 37°C, and was unable to grow at 48°C [35]. They concluded that any disorder in the encoding of *cgrA* in *A. fumigatus *can result in reduced thermotolerance and virulence [36]. 

Perlin et al. (2015) stated that one of the suggested mechanisms for acquiring azole resistance is related to the expression level of *cyp51A* genes [37]. Additionally, Mousavi et al. (2015) conducted an investigation by silencing *cyp51A* gene in azole‐resistant *A. fumigatus *straines. The authors revealed that the MIC of itraconazole for the treated cells reduced from 16 to 4 μg/mL, which was more noticeable than the change observed in untreated control cells [38]. Dąbrowska et al. (2015) found that the resistant strain harbors TR34/L98H mutation in the *cyp51A* gene and when cultured in media supplemented with voriconazole exhibits the overexpression of both *cyp51A* and *cyp51B* genes [39]. 

The aim of the present study was to evaluate the effect of fermented kombucha tea on the growth of azole-susceptible and -resistant *A. fumigatus *strains. In addition, the expression of *cgrA* and *cyp51A* genes was assessed in these strains. Our results demonstrated that kombucha fermented tea as a potent and natural antioxidant had inhibitory effects against the growth of azole-susceptible and -resistant* A. fumigatus* strains at the concentrations of 12,300-6,170 and 24,700 μg/mL, respectively. Furthermore, the transcription level of *cgrA* and *cyp51A* genes significantly reduced in both susceptible and resistant strains to 86.6% and 70.5% using 12,300 μg/mL and to 97.9% and 85.3% by 6,170 μg/mL kombucha, respectively. In addition, 24,700 μg/mL of kombucha diminished the transcription of susceptible and resistant strains to 84.3% and 84.6%, respectively. 

## Conclusion

The findings of the present study indicated kombucha fermented tea as a potent and powerful natural antioxidant at the examined specific concentrations. Our results also revealed that in vitro conditions can interfere with the pathogenesis of *A. fumigatus *by growth inhibition and reduction of gene expression. 

According to the previous studies conducted on animal models, kombucha tea has no significant toxicity. However, further studies are required to prove the lack of toxicity for this substance in the human cell line. In addition, it is recommended to perform further complementary studies to identify the antifungal properties of kombucha againt other important fungal pathogens. Moreover, the effect of other concentrations of kombucha metabolites needs to be investigated both in vitro and in vivo.

## Author’s contribution

S. R. and S. J. H. designed and managed the present research. S. Kh. performed real-time PCR and analyzed the data. L. N. performed the tests, analyzed real-time PCR data, and wrote the first draft of the manuscript. S. R. and S. Kh. edited the final manuscript. R. D. Gh. and M. S. were project partners. A. B. performed a critical review of the manuscript. All authors approved the final version of the manuscript.
